# Histiocytic Sarcoma Presenting as a Submandibular Mass in a 93-Year-Old Patient: A Case Report

**DOI:** 10.3390/reports9020161

**Published:** 2026-05-20

**Authors:** Evangelos Kostares, Athina Chatzigavriil, Georgia Kostare, Domna Efthymiou, Charikleia Kouvidou, Ourania Schoinohoriti, Christos Perisanidis, Stavroula Diamantopoulou

**Affiliations:** 1 Department of Oral and Maxillofacial Surgery, Dental School, National and Kapodistrian University of Athens, 115 27 Athens, Greece; 2 Department of Microbiology, Medical School, National and Kapodistrian University of Athens, 115 27 Athens, Greece; 3 Department of Pathology, Evangelismos General Hospital, Ipsilantou 45-47, 106 76 Athens, Greece

**Keywords:** histiocytic sarcoma, head and neck, lymph node, submandibular gland, case report, literature review

## Abstract

**Background and Clinical Significance**: Histiocytic sarcoma is a rare and aggressive hematopoietic malignancy, which is particularly uncommon in the head and neck region and exceedingly rare within lymph nodes associated with salivary glands. The present study aims to describe the clinical, radiologic, histopathologic, and immunophenotypic features of a primary histiocytic sarcoma, arising in a lymph node within the submandibular gland, and to highlight the diagnostic challenges and management considerations through a correlation with the existing literature. **Case presentation:** This case report was conducted according to the CARE guidelines. A 93-year-old male presented with a progressively enlarging mass at the right submandibular region. Clinical examination, magnetic resonance imaging, and fine-needle aspiration cytology were performed, raising suspicion for a malignancy. The patient underwent surgical excision of the right submandibular gland with limited level I_b_ lymph node dissection. Histopathological evaluation combined with an extensive immunohistochemical panel established the diagnosis of histiocytic sarcoma. The tumor was composed of pleomorphic epithelioid and spindle-shaped cells with marked cytologic atypia and high mitotic activity. Immunohistochemistry demonstrated strong positivity for histiocytic markers (CD163, CD68, CD14) and negativity for epithelial, lymphoid, and dendritic cell markers, allowing for the exclusion of major differential diagnoses. The proliferative index (Ki-67) was approximately 90%, indicating aggressive biological potential. FDG PET-CT performed two months after surgery showed no evidence of residual, regional, or distant disease. Considering the localized presentation and the patient’s advanced age, no adjuvant therapy was administered. During follow-up, no evidence of recurrence or disease progression was observed. **Conclusions:** Primary histiocytic sarcoma involving a lymph node within the submandibular gland is extremely rare and may clinically and cytologically mimic other malignancies. Accurate diagnosis relies on comprehensive immunohistochemical evaluation and exclusion of phenotypic mimickers. A review of previously reported cases of cervical lymph node histiocytic sarcoma demonstrated an age range from 35 to 80 years, with a male predominance and a higher incidence in Asian countries. Most cases presented with localized cervical lymph node disease. Surgical excision was the most commonly applied treatment, and was frequently associated with favorable outcomes, with several patients remaining disease-free during follow-up periods ranging from 24 to 48 months. The accumulation of additional well-documented cases is essential to improve diagnostic accuracy and guide evidence-based treatment strategies for this uncommon entity.

## 1. Introduction and Clinical Significance

Histiocytic sarcoma is a rare malignant hematopoietic neoplasm characterized by morphological and immunophenotypic features of mature histiocytes and classified among histiocyte/macrophage tumors in the current World Health Organization classification [1]. Its estimated incidence is extremely low, reported to be less than 0.17 cases per 1,000,000 individuals, and it typically demonstrates an aggressive clinical course with poor prognosis. The disease most frequently arises in extranodal sites of adults, including soft tissue, skin, the gastrointestinal tract, and the respiratory system, whereas lymph node involvement is less common [1,2]. Clinically, histiocytic sarcoma may present as a painless mass and can occur de novo or in association with other hematologic malignancies, further complicating diagnosis and management. Accurate diagnosis is challenging due to a significant morphologic overlap with other malignant neoplasms, making immunohistochemistry essential for confirmation [1,3]. Head and neck involvement is uncommon, and occurrence in submandibular lymph nodes is relatively rare [1,2,3,4].

The recent literature has also expanded the biologic understanding of histiocytic sarcoma. In a subset of cases, especially those arising in association with other hematolymphoid malignancies, shared molecular or cytogenetic abnormalities support a clonal relationship and suggest mechanisms such as transdifferentiation or lineage switching [1,2]. In addition, BRAF alterations have been reported in some histiocytic neoplasms, raising interest in the molecular pathogenesis of these tumors and in the possibility of targeted therapeutic approaches in selected patients [2,3].

Because of its rarity, broad clinicopathologic spectrum, and the lack of standardized treatment approaches, histiocytic sarcoma remains a challenging entity in both diagnosis and management. Consequently, the documentation of additional well-characterized cases is important to improve recognition of this uncommon neoplasm and to enrich the existing literature regarding its presentation, diagnostic features, biological behavior, and therapeutic options. Herein, we report a case of histiocytic sarcoma diagnosed after surgical excision of a submandibular mass in an elderly patient.

## 2. Case Presentation

This case report was prepared in accordance with the CARE guidelines.

A 93-year-old Greek male patient presented with a progressively enlarging swelling in the right submandibular region of approximately three months’ duration. The patient reported gradual increase in size without pain, dysphagia, or systemic symptoms such as fever, weight loss, or night sweats. His past medical history included a previously known prostate enlargement, for which he was not receiving active medical treatment. No regular medication use was reported.

**Figure 1:**
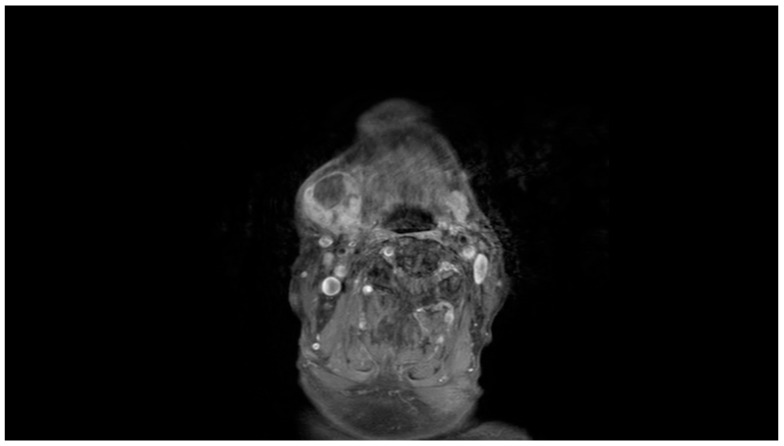
Coronal magnetic resonance image of the neck demonstrating a heterogeneous mass in the right submandibular region, involving the submandibular salivary gland and adjacent soft tissues.

**Figure 2:**
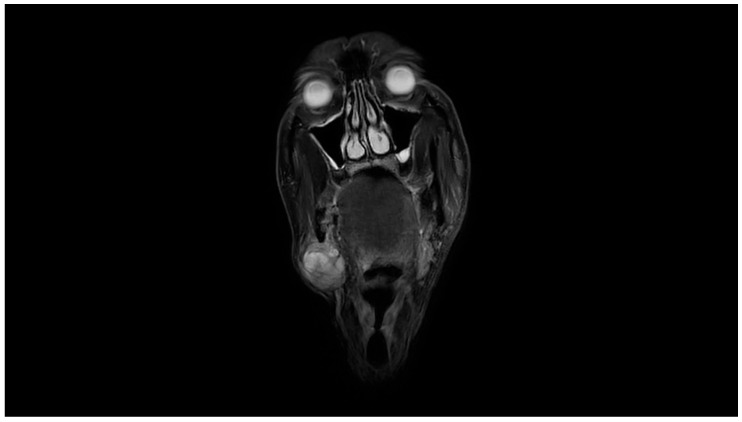
Axial contrast-enhanced magnetic resonance image of the neck demonstrating heterogeneous enhancement of the right submandibular lesion, with internal non-enhancing areas suggestive of necrotic change.

Clinical examination revealed a palpable mass in the right submandibular area, with no overlying skin changes or intraoral mucosal abnormalities. Facial nerve function was clinically intact. Magnetic resonance imaging (MRI) of the neck demonstrated a heterogeneous lesion in the right submandibular region, corresponding to a mass involving the submandibular salivary gland and adjacent soft tissues. The lesion measured approximately 3.6 × 3.4 × 3.4 cm and showed low signal intensity on T1-weighted images and heterogeneous signal intensity on T2-weighted images, with areas of intermediate signal intensity. Following intravenous contrast administration, the lesion demonstrated enhancement, with non-enhancing areas suggestive of necrotic changes. The imaging findings raised suspicion for a malignant neoplastic process involving the right submandibular gland region. No pathologically enlarged cervical lymph nodes were identified (Figure 1 and Figure 2).

Fine-needle aspiration biopsy (FNA) was subsequently performed and revealed numerous atypical neoplastic cells with pleomorphic nuclear features, suggestive of malignancy.

**Figure 3:**
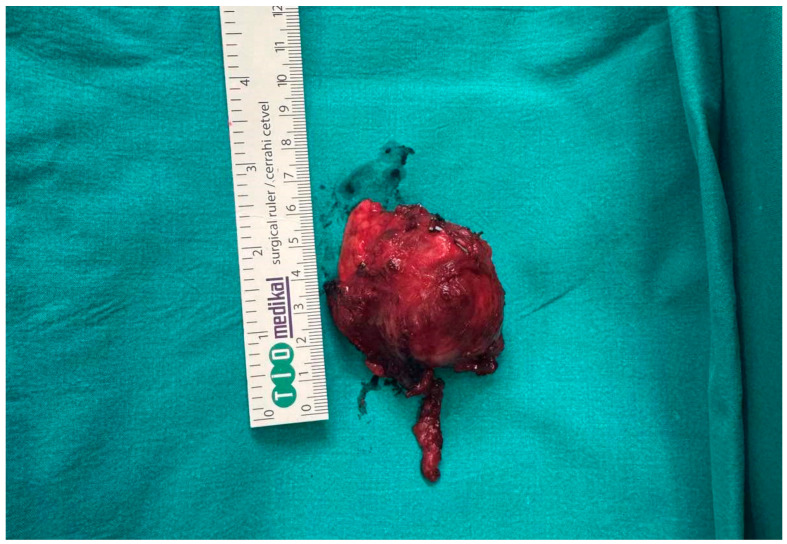
Gross photograph of the surgically excised specimen.

Given the imaging and cytological findings, the patient was scheduled for surgical treatment. Due to his advanced age, a conservative surgical approach was opted for. Under general anesthesia, a standard transcervical submandibular approach was performed through a horizontal cervical incision approximately 2 cm below the inferior border of the mandible. Subplatysmal flaps were elevated, and the marginal mandibular branch of the facial nerve was identified and preserved. The right submandibular gland was excised, along with a limited level Ib lymph node dissection (Figure 3).

**Figure 4:**
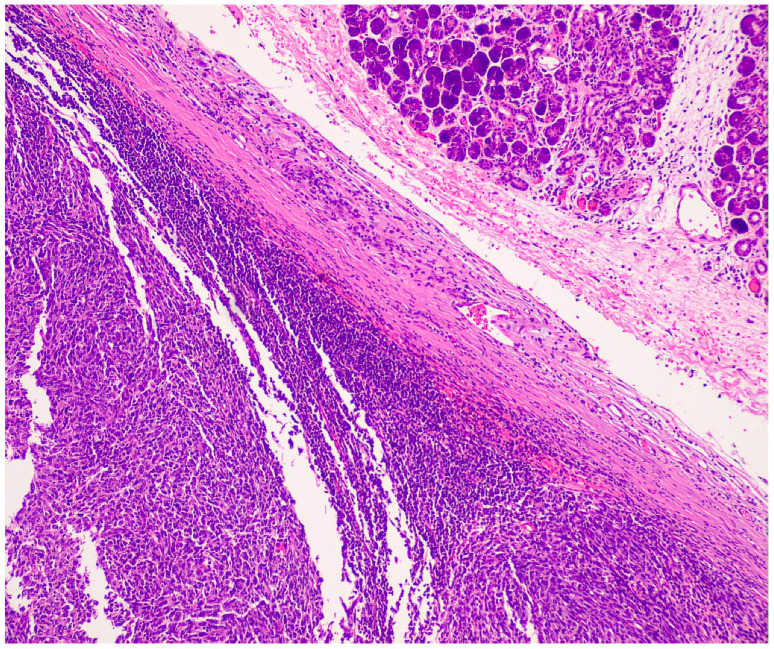
Circumscribed mass (left) and normal submandibular gland (upper right). H-E ×100.

**Figure 5:**
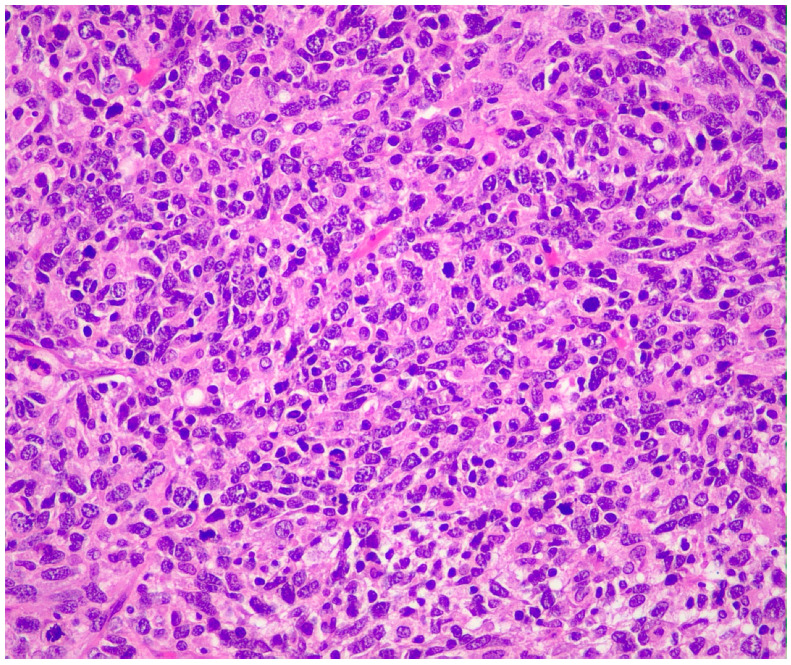
Large oval and spindle cells with atypical nuclei and high mitotic activity. H-E ×400.

Histopathological examination demonstrated a circumscribed malignant neoplasm measuring 3.0 × 2.6 × 2.5 cm, composed of large round-to-oval or spindle pleomorphic non-cohesive cells with atypical nuclei, variable prominent nucleoli, eosinophilic or amphophilic cytoplasm and high mitotic activity (Figure 4 and Figure 5).

**Figure 6:**
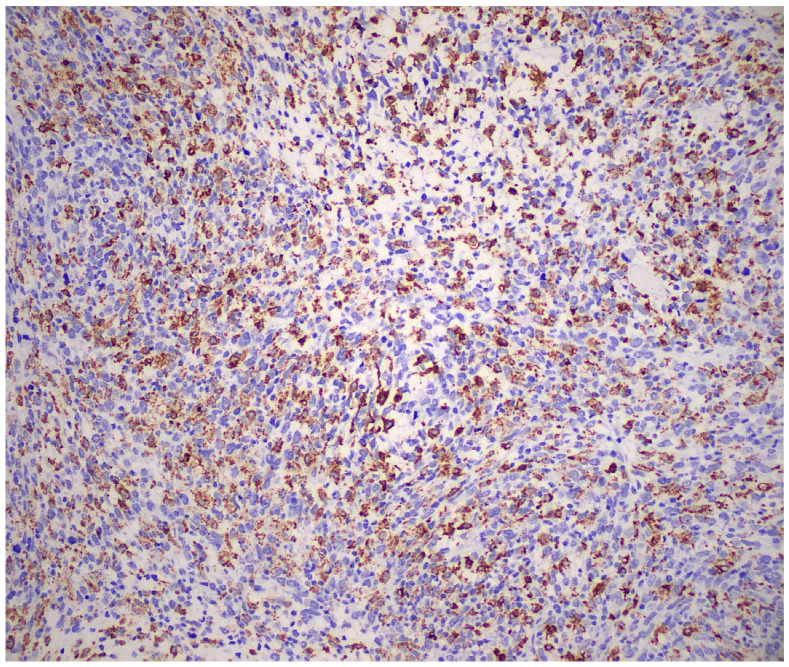
Neoplastic cells were positive for CD68 (×200).

**Figure 7:**
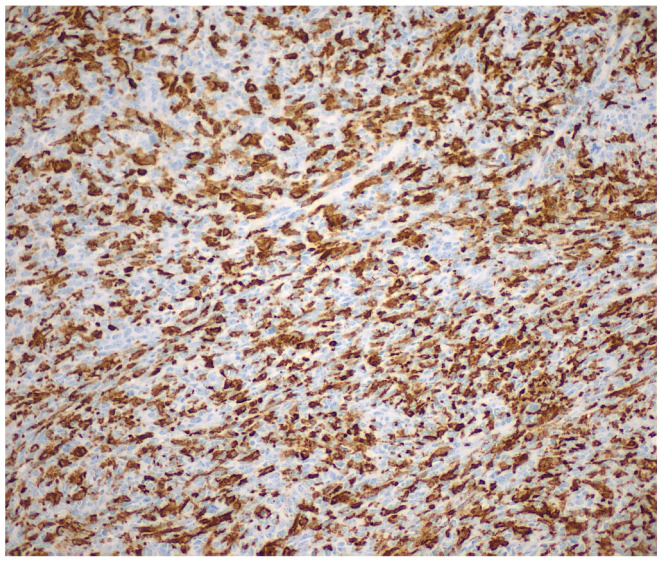
Neoplastic cells were positive for CD163 (×200).

**Figure 8:**
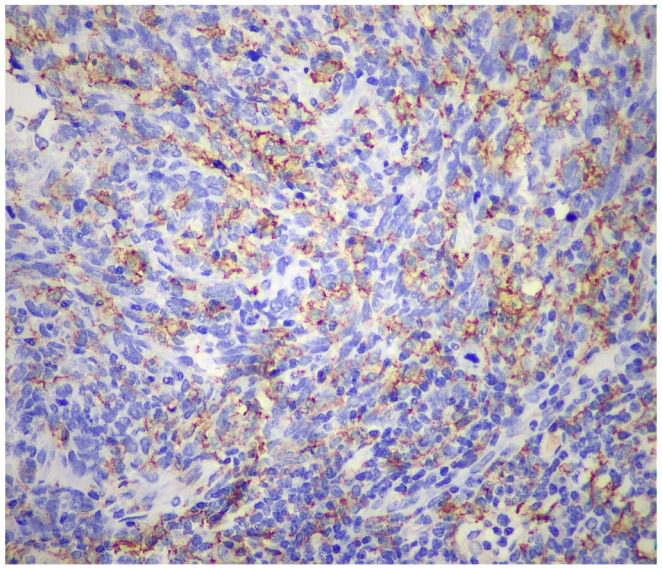
Neoplastic cells were positive for CD14 (×400).

Immunohistochemically, the neoplastic cells showed expression of multiple histiocytic markers, including CD68 (Figure 6), CD163 (Figure 7) and CD14 (Figure 8). It should be kept in mind that these markers are not specific for HS, and this is a diagnosis of exclusion. Tumor cells were negative for langerin, CD1a, ALK, OCT2 desmin, AE1/AE3, CK8/18, BerEP4, MOC-31, CD34, caldesmon, BRAF, p40, CK7, S-100, SOX-10, p63, beta-catenin, CD21, CD23, and CD35. The expression of INI-1 was preserved. EBER was negative. There was high proliferative activity (Ki-67 approximately 90%). Figure 4, Figure 5, Figure 6, Figure 7 and Figure 8 illustrate the key immunohistochemical findings supporting histiocytic differentiation of the neoplastic cells, namely positivity for CD68, CD163, and CD14, which were interpreted together with the broad negative immunohistochemical panel to establish the diagnosis of histiocytic sarcoma. The immunohistochemical panel was used to exclude the main differential diagnoses. Negativity for epithelial markers supported exclusion of poorly differentiated carcinoma; negativity for langerin and CD1a excluded Langerhans cell sarcoma; negativity for CD21, CD23, and CD35 excluded follicular dendritic cell sarcoma; and negativity for S-100 and SOX-10 argued against melanoma. In addition, ALK negativity and the absence of supportive lymphoid marker expression helped exclude anaplastic large-cell lymphoma.

The above immunohistochemical findings excluded the histological differential diagnosis of HS from anaplastic large cell lymphoma (ALCL), Langerhans cell sarcoma (LCS), interdigitating dendritic cell sarcoma (IDCS), follicular dendritic cell sarcoma (FDCS), myeloid sarcoma, melanoma, undifferentiated large-cell carcinoma, epithelioid sarcoma, and undifferentiated pleomorphic sarcoma (UPS). The surgical margins were found to be clear (R0).

**Figure 9:**
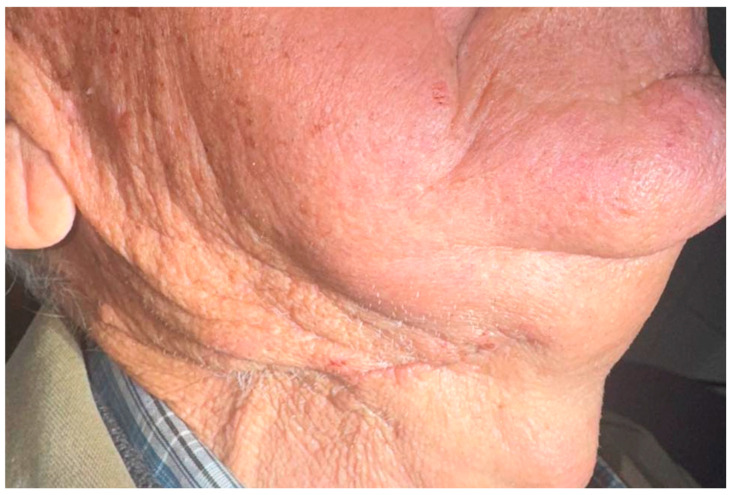
Postoperative clinical appearance following excision of the right submandibular mass, showing satisfactory wound healing without evidence of local complications.

The postoperative course was uneventful (Figure 9), with no evidence of surgical site infection, facial nerve dysfunction, hematoma, or other complications. FDG PET-CT performed approximately two months after surgery revealed no evidence of residual disease, regional spread, or distant metastasis. In view of the localized disease and the patient’s advanced age, no adjuvant therapy was administered. This decision was discussed in a multidisciplinary setting and was also influenced by the patient’s preference, as he did not wish to undergo additional postoperative therapy.

To date, the patient has been followed for 13 months after surgery and remains under ongoing surveillance. Surveillance consisted of monthly clinical examination, MRI of the neck and facial skull, and hematological evaluation every three months. In addition, FDG PET-CT performed approximately two months postoperatively showed no evidence of residual disease, regional involvement, or distant metastasis. At the most recent follow-up, 13 months postoperatively, no recurrence was identified clinically or radiologically, and no evidence of systemic disease progression was detected.

## 3. Discussion

The histiocytic sarcoma represents less than 1% of hematolymphoid malignancies and only a very small proportion of head and neck tumors, making diagnosis particularly challenging in this anatomical region. Primary involvement of lymph nodes located within or adjacent to the submandibular gland is exceedingly uncommon, with only isolated cases described in the literature, highlighting the rarity of the present case.

Clinically, histiocytic sarcoma demonstrates a wide age distribution, although most of the reported cases affected middle-aged adult patients. Nevertheless, cases occurring in elderly individuals have been documented, indicating that advanced age does not preclude diagnosis. Patients typically present with a painless enlarging cervical mass, often without systemic “B” symptoms, especially in localized disease. Similar to our patient, Kubota et al. (2022) [5] described an 80-year-old male presenting with a slowly enlarging submandibular-region mass originating from a neck lymph node, with imaging findings suggestive of malignancy but lacking specific radiologic characteristics. Imaging modalities such as CT, MRI, and PET-CT are useful for staging but do not provide diagnostic specificity, and histopathological examination remains essential. The patient was treated with surgical excision and neck dissection without adjuvant therapy, achieving disease-free survival at two years, supporting the role of surgery as an effective treatment option in localized disease. A similar observation was reported by Oka et al. (2021) [6], who described a 74-year-old male with a cervical mass that was incidentally identified as histiocytic sarcoma concomitantly with laryngeal squamous-cell carcinoma. After surgical resection of the cervical lesions, the patient received systemic chemotherapy with the CHOP regimen (cyclophosphamide, doxorubicin, vincristine, and prednisolone). Post-treatment PET/CT demonstrated complete remission, and the patient remained disease-free during four years of follow-up.

Morphologically, histiocytic sarcoma is characterized by diffuse proliferation of large pleomorphic epithelioid or spindle-shaped cells with abundant eosinophilic cytoplasm, prominent nucleoli, and marked nuclear atypia. Hemophagocytosis and necrosis may also be observed [7]. As reported by Mallya et al. (2014) [8], histiocytic sarcoma can cytologically mimic poorly differentiated carcinomas or lymphomas, leading to frequent initial misclassification. Therefore, adequate tissue sampling and extensive immunohistochemical analysis are critical for accurate diagnosis.

Because of its rarity, there are no standardized therapeutic guidelines for the management of histiocytic sarcoma. Surgical excision remains the preferred treatment for localized lesions, while chemotherapy and radiotherapy are generally reserved for disseminated disease, unresectable disease or positive surgical margins. Conventional lymphoma-based regimens such as CHOP have been used with variable success, and emerging targeted therapies and immunotherapeutic approaches are under investigation. In elderly patients, treatment decisions must also take into consideration comorbidities and functional status. In our case, complete surgical excision followed by close surveillance was selected, due to the localized disease and the patient’s advanced age, a strategy supported by previously reported favorable outcomes in solitary nodal histiocytic sarcoma [1,9].

A review of the PubMed database was conducted using relevant MeSH terms and keywords up to 9 March 2026. The review of the previously reported cases of cervical lymph node histiocytic sarcoma showed that patients ranged in age from 35 to 80 years, with a predominance of male patients, and that most cases were reported from Asian countries. The majority presented with localized cervical lymph node disease. Surgical excision was the most commonly applied treatment and was frequently associated with favorable outcomes, as all surviving patients remained disease-free during follow-up periods ranging from 24 to 48 months (Table 1).

A major strength of this case report lies in the documentation of an exceptionally rare presentation of histiocytic sarcoma arising within a lymph node associated with the submandibular gland, supported by comprehensive clinical, radiologic, histopathologic, and extensive immunohistochemical evaluation, which allowed reliable exclusion of important differential diagnoses. The detailed diagnostic work-up and correlation with previously reported cases contribute valuable data to the limited literature and help improve awareness of this uncommon entity among head and neck clinicians and pathologists. However, several limitations should be acknowledged. As a single-case observation, the findings cannot be generalized and do not allow conclusions to be made regarding optimal treatment strategies or prognosis. In addition, the relatively short follow-up period limits assessment of long-term disease behavior, particularly given the aggressive biological potential suggested by the high proliferative index. Another limitation is that PD-L1 immunohistochemical testing was not performed; therefore, the potential relevance of immune checkpoint inhibitor therapy could not be assessed in this case. Finally, treatment decisions were influenced by the patient’s advanced age, which may restrict comparison with outcomes reported in younger populations. Nevertheless, the report adds meaningful evidence to the existing body of knowledge and highlights the need for accumulation of further well-documented cases to guide evidence-based management.

## 4. Conclusions

Histiocytic sarcoma in the head and neck region is extremely rare, frequently mimics other malignancies, and requires comprehensive immunohistochemical evaluation for diagnosis. Continued accumulation of well-documented cases is essential to better define prognostic factors and establish evidence-based therapeutic strategies for this uncommon malignancy.

## Figures and Tables

**Table 1:** Literature review of histiocytic sarcoma involving cervical lymph nodes.

First Author	Year of Publication	Continent of Origin	Country	Gender	Age (Years)	Dimensions (cm) (Histopathological)	Disease Extent	Treatment	Follow-Up (Months)	Status
Our case	2026	Europe	Greece	Male	93	3 × 2.6 × 2.5	Localized right submandibular/cervical nodal disease	Surgical resection	13	Alive
Kottangal G.V. [10]	2025	Asia	India	Female	60	NA	Multifocal nodal disease	Palliative care	-	-
Kubota A. [5]	2022	Asia	Japan	Male	80	3.8 × 3.8 × 2.6	Solitary localized right cervical/submandibular nodal disease	Surgical resection	24	Alive
Oka S. [6]	2021	Asia	Japan	Male	74	1.1 × 0.8	Localized cervical disease, concomitant with laryngeal squamous cell carcinoma	Surgical resection and chemotherapy	48	Alive
Kim J.M. [7]	2016	Asia	Korea	Male	55	3 × 2.5 × 2 (CT)	Localized left supraclavicular nodal disease	Surgical resection	10	Died of disease
Mallya V. [8]	2014	Asia	India	Male	35	NA	Localized left cervical lymph node disease	-	-	-
Miliauskas J.R. [9]	2003	Australia	Australia	Female	52	NA	Localized left upper cervical nodal disease	Chemotherapy	36	Alive

NA: not applicable.

## Data Availability

The data supporting the findings of this case report are available within the article. Additional data are not publicly available due to patient privacy and ethical restrictions.
